# Sinus Mucosa Thinning and Perforations after Sinus Lifting Performed with Different Xenografts: A Histological Analysis in Rabbits

**DOI:** 10.3390/dj10010002

**Published:** 2021-12-28

**Authors:** Riccardo Favero, Karol Alí Apaza Alccayhuaman, Daniele Botticelli, Samuel Porfirio Xavier, Vitor Ferreira Balan, Veronica Macchi, Raffaele De Caro

**Affiliations:** 1 Dipartimento di Neuroscienze, Università di Padova, 35128 Padova, Italy; rickyfavero@msn.com (R.F.); veronica.macchi@unipd.it (V.M.); raffaele.decaro@unipd.it (R.D.C.); 2 Department of Oral Biology, Medical University of Vienna, 1090 Vienna, Austria; 3 ARDEC Academy, 47923 Rimini, Italy; daniele.botticelli@gmail.com; 4 Department of Oral and Maxillofacial Surgery and Periodontology, Faculty of Dentistry of Ribeirão Preto, University of São Paulo, São Paulo 14040-904, Brazil; spx@forp.usp.br (S.P.X.); vitor.balan@usp.br (V.F.B.)

**Keywords:** animal study, sinus floor elevation, bone healing, Schneiderian membrane, histology

## Abstract

Background: Experimental studies have shown a progressive thinning and perforations of the sinus mucosa associated with sharpened edges and the cutting projections of graft particles used simultaneously for maxillary sinus augmentation. Hence, the aim of the present study was to evaluate the damaging effects of two different bovine grafts on the sinus mucosa after sinus augmentation. Methods: Twenty New Zealand rabbits received a bilateral sinus lifting using, as fillers, two different types of deproteinized bovine bone in granules, one processed at low temperature (low-T group), and the other at high temperature (high-T group). Thinned mucosa sites (<40 µm) and perforations were evaluated in the sinus mucosa that were in contact with graft granules after 2 and 10 weeks, in ten animals per period. Results: After 2 weeks of healing, the number of thinned mucosa sites was 118 in the low-T group, and 149 in the high-T group (*p* = 0.191). At the 10-week assessment, the thinned sites increased to 237 and 195 sites, respectively. The numbers of sinus mucosa perforations after 2 weeks were eight and three in the low-T and high-T group, respectively. At the 10-week evaluation, the perforations increased to 19 in the low-T group, and to 14 in the high-T group. Conclusions: The contact with bovine xenografts yielded thinning and perforations of the sinus mucosa. Despite the differences in characteristics and dimensions, no differences were found between the two xenografts in the numbers of thinning mucosa sites and perforations. However, a trend of more events was found in the low-T compared to the high-T group.

## 1. Introduction

To increase the volumes in the posterior segment of the maxilla, a sinus floor augmentation procedure is often applied to allow the placement of dental implants with the aim of rehabilitating that region. This technique has been proven to have a high success rate [1,2]. One of the most common complications during surgery is the perforation of the sinus mucosa [3,4]. Perforations have also been observed during the placement of biomaterial or implants in the subantral space after sinus mucosa elevation [5,6,7]. Even though these perforations might heal spontaneously [6], sinusitis has been associated with extruded biomaterial [8,9,10], an event that requires the removal of the graft remnants from the sinus cavity [9,10]. 

Perforations have also been observed over time, triggered by both biomaterials and implants. In experiments on maxillary sinus lifting in rabbits [11,12,13], a progressive thinning of the sinus mucosa and a progressively increased number of perforations were observed over time. Thinning mucosa and perforations were seen on sharpened edges and cutting projections of grafts made of deproteinized bovine bone mineral (DBBM) [11,12]. However, when autogenous bone was used as filler, no perforations, and few thinning mucosa sites, were observed after 7 and 40 days of healing [11]. Thinning sinus mucosa regions and perforations were also reported on implant apex and threads in experiments in rabbits [11,13]. 

The characteristics and rate of resorption varies for different grafts [11,14] and the dimensions of the graft granules might influence the number of sinus mucosa thinned sites and perforations [12]. It seemed rational to perform studies aiming to evaluate differences in the effects of various grafting biomaterials on the sinus mucosa. Hence, the aim of the present study was to evaluate the damaging effects of two different bovine grafts on the sinus mucosa after sinus augmentation.

## 2. Materials and Methods

### 2.1. Ethical Statements

The experimental protocol was submitted to and approved by the Ethical Committee of the Faculty of Dentistry of Ribeirão Preto, University of São Paulo (protocol No 2019.1.113.58.1; 8 April 2019). The article was written according to the ARRIVE guidelines. The Brazilian guidelines for animal care were accurately followed.

### 2.2. Study Design

A split-mouth design was adopted. Maxillary sinus augmentation was carried out bilaterally in twenty rabbits. Deproteinized bovine bone grafts of different characteristics were randomly placed one per sinus.

The number and width of sites presenting sinus mucosa reduced in dimensions and the number of perforations of the sinus mucosa in contact with the filler material were assessed. The histomorphometric data describing the healing within the elevated region were reported elsewhere [15].

### 2.3. Experimental Animals

Twenty albino New Zealand rabbits, 3.5–4 kg in weight and 4–5 months old, were used for the experiment. Two groups were obtained, ten animals each, and euthanized after 2 or 10 weeks from surgery, respectively.

### 2.4. Biomaterials

Two biomaterials processed at different temperatures were used.

Low-T (low temperature): Bio-Oss^®^ (granules 0.250–1.0 mm; Geistlich Biomaterial, Wolhusen, LU, Switzerland) is a natural inorganic, porous hydroxyapatite from bovine cancellous bone obtained through a deproteinization process at a temperature of 300 °C. Bio-Oss^®^ has high porosity (70.5% to 80%), with pores of 20 to 200µm, a low level of hydrophilicity, low crystallinity and an amorphous structure [16,17].

High-T (high temperature): Cerabone^®^ (granules 0.5–1.0 mm; Botiss Biomaterials GmbH, Zossen, Germany) is composed of a ceramic hydroxyapatite (pentacalcium hydroxide trisphosphate) from bovine cancellous bone produced in a high-temperature process (>1200 °C). Cerabone^®^ possesses a foam-like surface structure with porosity of 65% to 80%, pores of 600 to 900 µm, high hydrophilicity and high crystallinity, with very low levels of impurities [16,17].

### 2.5. Sample Size

The sample was determined for the histomorphometric study performed on the same experiment [15]. Samples comprised 10 animals per group to make it possible to find differences in statistical significance in bone formation within the elevated space between the experimental groups. For the present study, two experimental articles were taken into consideration. In one study [18], it was shown that the particle size of high-T granules was 2.7 times larger than low-T granules. Moreover, in another study in which six animals were used for each period of evaluation [12], after 8 weeks of healing, the number of perforations of the sinus mucosa was double at the smaller (0.125–1.0 mm) compared to the larger (1–2 mm) granules of a DBBM graft. Considering that, also in the present study, the biomaterials used have different sizes, one smaller than the other, ten animals were considered sufficient to disclose differences in number of perforations of the sinus mucosa.

### 2.6. Randomization and Allocation Concealment

The randomization plan was created electronically on 10 May 2019 at randomization.com by an author (S.P.X.) who was not involved in the surgeries. The treatment assignments were held in opaque sealed envelopes. After the sinus mucosa was elevated bilaterally, the envelopes were opened and the treatment assignment was disclosed to the surgeon (V.F.B.). The assessor of the histological slides (K.A.A.A) could not be blind because the two biomaterials acquired different colors after staining.

### 2.7. Clinical Procedures

**Figure 1:**
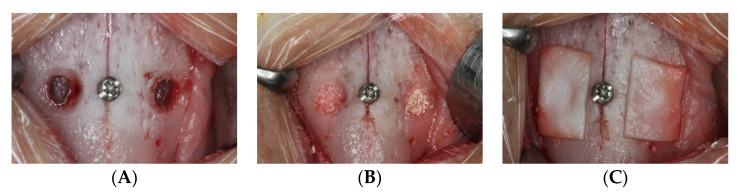
Clinical overview of the surgical procedures: (**A**) antrostomy preparation (**B**), elevated space filled with grafts (**C**), collagen membrane covering the antrostomies.

The anesthetic procedures included the use of 1.0 mg/kg of acepromazine (Acepran^®^, Vetnil, Louveira, São Paulo, Brazil) subcutaneously and a mix of 3.0 mg/Kg xylazine (Dopaser^®^, Hertape Calier, Juatuba, Minas Gerais, Brazil) and 50 mg/kg of ketamine hydrochloride (Ketamin Agener, União Química Farmacêutica Nacional S/A, Embu-Guaçú, São Paulo, Brazil) administered intramuscularly. After shaving and disinfection of the region, an incision of the midline of the nasal dorsum was performed by an expert surgeon (V.F.B.). The nasal bone was exposed and two ostetomies were prepared bilaterally of the nasal-incisal suture using a trephine (Neodent, Curitiba, Paraná, Brazil; 4.1 and 3.4 mm external and internal diameters, respectively) and a round diamond drill (Figure 1A). A small screw was placed in the nasal-incisal suture as reference for the histological process. The sinus mucosa was elevated with an elevator of small dimensions (718-EN1; Bontempi Strumenti Chirurgici, San Giovanni in Marignano, RN, Italy) and similar quantity of grafts were placed in the elevated spaces. (Figure 1B). The osteotomies were protected with membranes in collagen (Figure 1C; Geistlich Biomaterial, Wolhusen, LU, Switzerland).

### 2.8. Euthanasia

The rabbits were first anesthetized and then euthanized with carbon dioxide (CO_2_).

### 2.9. Housing and Husbandry

The animals were kept in individual cages in a climatized room with access to food and water ad libitum. The biological functions and the wounds were checked daily by specialized operators for the whole period of the experiment.

### 2.10. Histological Preparation

Biopsies of the experimental regions were collected, fixed in formalin and then dehydrated and included in resin (LR White™ hard grid, London Resin Co Ltd., Berkshire, UK) and polymerized. Two ground sections were obtained using cutting and grinding equipment (Exakt^®^, Apparatebau, Norderstedt, Germany) and stained with either Stevenel’s blue and alizarin red or toluidine blue.

### 2.11. Calibration for Histometric Evaluations

A well-trained assessor made the histological evaluations (K.A.A.A.). The intra-rater reliability in the measurements of the sinus mucosa width and perforation dimensions was K > 0.90.

### 2.12. Histological Analyses

The pristine mucosa was measured at the medial and lateral sinus walls in regions not included in the elevated area. A mean value of the two measurements was used for analysis.

The number and width of the elevated sinus mucosa in close contact to the graft granules was measured, and all measurements <40 µm were recorded. The number and dimensions of the sinus mucosa perforations at the graft granules were assessed.

### 2.13. Experimental Outcomes and Statistical Methods

Prism 9.1.1 (GraphPad Software, LLC, San Diego, CA, USA) was used for statistical analyses. The normal distribution of the data was assessed with the Shapiro–Wilk test for both paired and unpaired variables. Either a paired t test or a Wilcoxon test was used to evaluate differences between the low-T and high-T group. Differences between the two periods of healing were evaluated using either an unpaired t test or a Mann–Whitney test.

## 3. Results

### 3.1. Clinical Outcomes

One sinus mucosa of the low-T group in the 2-month period presented a small perforation during surgery and was covered with a collagen membrane. The collagen membrane, still present after 2 weeks, was hindering a large part of the mucosa from any contact with the biomaterial granules. For this reason, this sinus was excluded from analysis together with the contralateral sinus. No animals presented problems during healing; thus, the sample size was *n* = 9 for the 2-week period, and *n* = 10 for the 10-week period.

### 3.2. Descriptive Histological Evaluation

**Figure 2:**
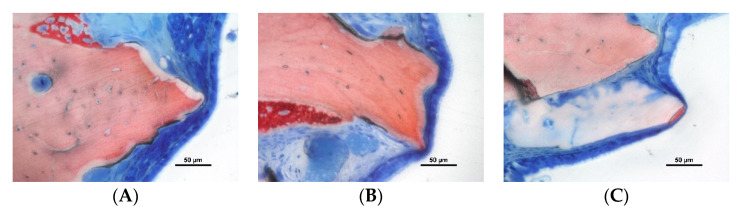
Photomicrographs of ground section of low-T sites: (**A**) vessels and mucous glands dislocated by the granules; (**B**) thinning of the epithelial cells; (**C**) tapered epithelial cells adjacent a very thin remnant of sinus mucosa. Stevenel’s blue and alizarin red stain.

**Figure 3:**
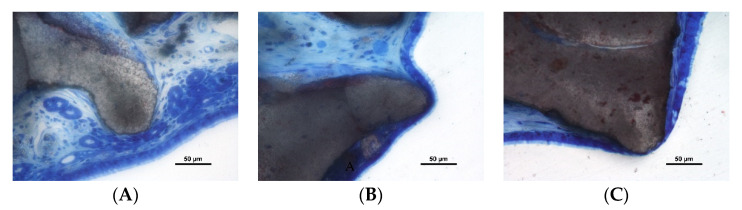
Photomicrographs of ground section of high-T sites: (**A**) vessels and mucous glands dislocated by the granules; (**B**) thinning of the epithelial cells still presenting cilia; (**C**) tapered epithelial cells adjacent a very thin remnant of sinus mucosa. Stevenel’s blue and alizarin red stain.

More than one hundred thinned mucosa sites (<40 µm) were found in both groups, and the number increased at the 10-week compared to the 2-week evaluation. The thinned mucosa sites were associated with granules, mainly at surfaces protruding beyond the dome profile of the elevated spaces. The mucosa was found in tight contact with the granules’ surface and the tissues contained in the submucosa were affected by this contact. The degree of damage showed a progressive trait. In the first stages, a dislocation of vessels and mucous glands was observed, while the pseudostratified epithelium was not affected yet (Figure 2A and Figure 3A). However, at the thinnest sites, the epithelium became involved in the damage, presenting a progressive decrease in width and a loss of cilia and globet cells (Figure 2B and Figure 3B). Finally, only a very thin layer of soft tissues was laying on the graft surface (Figure 2C and Figure 3C).

**Figure 4:**
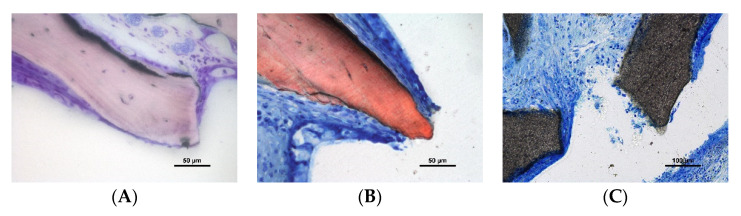
Photomicrographs of ground sections. Perforations of the sinus mucosa. Note the tapered shape of the epithelium in a tentative attempt to protect the subjacent submucosal tissues. (**A**,**B**) Inflammatory cells were rare; (**C**) inflammatory reaction—note the epithelium trying to isolate the granule on the left side of the image. (**A**) Toluidine blue stain; (**B**,**C**) Stevenel’s blue and alizarin red stain.

**Figure 5:**
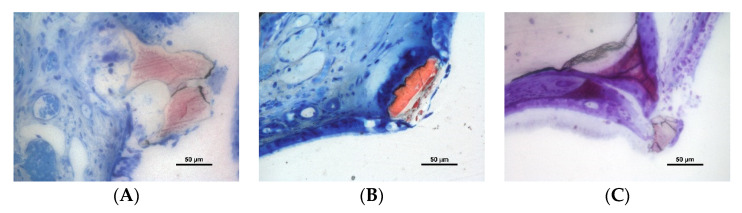
Photomicrographs of ground sections. Granules of low-T granules at the time of being expelled from the elevated space through the sinus mucosa. (**A**,**B**) Stevenel’s blue and alizarine red stain; (**C**) toluidine blue stain.

Perforations of the sinus mucosa were observed in both periods and biomaterials (Figure 4A–C and Figure 5A–C). Granules were found trespassing the sinus mucosa and the perforations were bordered by a tapered epithelium. In some instances, the regions presented none or few inflammatory cells (Figure 4A,B). However, in other cases, an inflammatory infiltrate was encompassing the granules (Figure 4C).

Some granules were found expelled through the sinus mucosa (Figure 5A–C).

### 3.3. Histometric Assessments

The width of the pristine sinus mucosa was similar in both groups and periods (mean values ranged between 54 µm and 61; Table 1). After 2 weeks of healing, in the low-T group, the sites of the elevated sinus mucosa presenting a width < 40 µm numbered 118, eleven of which were <10 µm (Table 2). The minimum width registered was 5 µm. Eight perforations were found in the low-T group in six sinuses. The largest perforation was about 2.4 mm in dimension, while five perforations were between 100 and 500 µm, and two were <100 µm. 

In the high-T group, after 2 weeks of healing, 149 sites presented a mucosa width < 40 µm, 16 of which were <10 µm, and 2 µm was the lowest value registered. Three perforations in three sinuses were found, presenting dimensions of about 700 µm, 300 µm, and 40 µm, respectively. In the 2-week period, no statistically significant differences were found between groups for any variable analyzed.

After 10 weeks of healing, in the low-T group, all sinuses presented thinned mucosa, reaching 237 sites in total (Table 1). The mean width was 19 µm and 46 sites were <10 µm, with the lowest being 2 µm (Table 2). Nineteen perforations, distributed among nine sinuses, were found in this period of healing. Six of these perforations presented dimensions between 100 and 200 µm, while the remaining thirteen were <100 µm, of which seven were <50 µm.

In the high-T group, after 10 weeks of healing, 195 thinned sites were found in ten sinuses. The mean width was 21 µm, and 44 sites presented a width < 10 µm, with 2 µm being the lowest width observed. Fourteen perforations were seen in seven sinuses, nine of which exhibited dimensions between 100 and 700 µm, and five were < 100 µm. In the 10-week period, no statistically significant differences were found between groups for any variable analyzed.

The differences between the width of the pristine and thinned mucosae were statistically significant for both groups and periods. Considering the difference between periods, only the width of the thinned mucosa and the number of thinned sites of the low-T xenograft were statistically significant.

## 4. Discussion

The aim of the present experiment was to assess the damaging effects of two bovine grafts on the mucosa of the sinus subsequently sinus augmentation. Both xenograft granules yielded damage to the sinus mucosa, dislodging the soft tissues in the submucosa region, decreasing the mucosa thickness associated with the loss of cilia and, finally, perforating the sinus mucosa. The number of thinned sites (<40 µm in width) and perforations increased over time. The differences between the two biomaterials, in relation to porosity, pore dimensions and hydrophilicity levels, did not yield statistically significant differences for the variables analyzed. This, in turn, means that these differences did not significantly influence sinus mucosa thinning and perforations. However, a tendency of presenting more thinning sites and perforations was detected for the low-T group compared to the high-T group. The reason for this tendency might be attributed to the larger size of the high-T graft compared to the low-T [18] that increased the frequency of contacts between the grafts and the sinus mucosa.

After 2 weeks of healing, six perforations in three sinuses in the low-T group, and eight perforations in three sinuses in the high-T group, were observed. It could be speculated that these perforations might have been procured, and not recognized, during surgery, or generated by the pressure applied to the biomaterial during the filling of the elevated space. Indeed, perforations of the sinus mucosa during the surgical procedures have been documented both in human [9,10] and in ex-vivo [8,19,20] studies. Moreover, in the present study, one perforation was observed during elevation and protected with a collagen membrane. However, the number of perforations increased over time, and this indicates against mucosal damage that was only related to the surgical injury. Moreover, the dimensions of the perforations after 2 weeks appeared to be larger than that after 10 weeks. In the low-T group, in the two-week period, eight perforations were found, of which one measured 2.4 mm, five measured between 100 and 500 µm and two measured < 100 µm. In the same group, after 10 weeks, all nineteen perforations were <200 µm. It might be hypothesized that the largest perforations of the 2-week period could be ascribed to surgical trauma. However, the smallest perforations, and those encountered at the 10-week period, are more likely to have occurred over time, through the progressive thinning and injuring processes at the sinus mucosa discussed above. In the high-T group, the perforations increased in number over time as well. However, large dimensions of the perforation were found also in the 10-week period. The difference with the low-T group in terms of dimensions might be related to the size of the granules, with those of the high-T group being 2.7 times larger than the low-T ones [18]. 

Reparative processes were observed in the soft tissues at the perforations. However, these processes appeared to be a tentative attempt at circumscribing the granules and, eventually, expelling them outwards towards the elevated space instead of trying to keep them in.

The decreased width of the sinus mucosa over time also provides support to the notion that there was a progressive increase in number of perforations. Again, it might be claimed that the mucosa thinning might have happened during surgery. However, damage such as the displacement of tissue components, the progressive decrease in width of the submucosa and epithelial layers, and the loss of cilia cannot be procured by the pressure applied to the biomaterial during the filling of the elevated space. The thinned sites increased in number over time, and this indicates once again in favor of progressive damage to the sinus mucosa having occurred. Between 2 and 10 weeks of healing, the thinned mucosa increased by >100 sites in the low-T group, and by about 50 sites in high-T group. Moreover, in the 10-week period, 44–46 thinned sites presented a width < 10 µm.

The outcomes obtained in the present study completely agree with those reported by other studies in rabbits that disclosed an increased number of perforations over time, and hundreds of thinned mucosae sites [11,12,13]. In one of these studies [12], sinus augmentation was performed in eighteen rabbits using DBBM granules of either 0.250–1.0 mm (small group) or 1–2 mm (large group) in dimension. The healing was studied after 2, 4 and 8 weeks, with six animals each group. Between 2 and 8 weeks, the thinned sites increased from 52 to 59 in the large group, and from 55 to 74 in the small group. The perforations increased from 1 to 5 in the large group, and from 0 to 8 in the small group. These outcomes correspond to those detected in the present experiment in which the number of thinned mucosae and perforations increased to a higher extent in the group with the smallest granules (low-T) compared to the group with the largest granules (high-T).

In another similar study in rabbits [11], the sinuses were augmented with either a DBBM or autogenous bone, and implants were placed simultaneously. The healing was studied after 7 and 40 days in twelve rabbits (six animals per period). In the DBBM group, between the two periods, the thinned mucosa sites in contact with the granules increased in number from 59 to 96 while, in the autogenous group, the thinned sites decreased from 14 to one. After 40 days, three perforations were observed in the DBBM group, while none in the autogenous group. However, in the 40-day period of healing, owing to the different rates of resorption of the two biomaterials, the sinus mucosa came into contact with the apex and threads of the implants to a higher extent in the autogenous compared to the DBBM group. This condition yielded three perforations at implants in two sinuses in the autogenous group, and only one perforation in one sinus in the DBBM group, while no perforations at implants were registered after 7 days of healing in any group. This, in turn, means that a biomaterial with a low rate of resorption protects the sinus mucosa from contact with the implant apex and threads. However, at the same time, this low resorption rate exposes the sinus mucosa to thinning and perforations caused by the graft itself. From a clinical point of view, however, it should be considered that the corticalization of the new sinus floor might protect against further perforations. In fact, total or partial corticalization of the new floor was documented in 30% to 75% of cases after 9 months of healing [21,22,23,24]. Moreover, sinus mucosa perforations might be restored with the expulsion of the granules from the elevated space, while a similar outcome is not desirable for implants.

Possible sinus mucosa perforations at implants were addressed in an experiment in sixteen rabbits [13]. Implants, with the surface of the test implants exposed to an argon plasma treatment, were simultaneously placed in augmented sinuses without fillers. After eight weeks of healing, the sinus mucosa was found collapsed onto the implants’ apexes and threads. This tight contact triggered sinus mucosa perforations at the apexes and threads of twenty-six out of thirty-two implants. It should be noted that perforations of the sinus mucosa were also reported for resorbable [25,26] and non-resorbable [27] devices used to keep the sinusal mucosa elevated during healing.

The collapse of the sinus mucosa at the top of biomaterials and implants might be explained by the tendency of the sinus to return to its original dimensions, as documented by experimental [28,29,30,31,32] and clinical studies [21,22,23,24]. The sinus mucosa will adapt its shape to that of hard tissues and implants. In the first period of healing, the post-surgical edema/bleeding will keep the sinus mucosa away from granules and implants [33]. In the present study, after two weeks, the protective action of the edema was concluded and the sinus mucosa was already adapted to the shape of the surface of the elevated space, resulting in a high number of thinned mucosa sites.

The limitation of the present study is mostly related to the model used, which presented a thinner width of the sinus mucosa compared that in humans [34,35], which was, therefore, more prone to damage caused by the graft granules. Another limitation is that the analysis was performed in only two histological slides that represented only the central region of the elevated space. An analysis of the whole surface of the elevated mucosa might have disclosed many more damaged sites. Other limitations of the present study are the faster rate of healing compared to humans [36] and the short periods of healing assessed, which did not allow a complete corticalization of the new sinus floor. Different biomaterials should be tested with the aim of finding those producing the lowest damage to the sinus mucosa.

The results from the present study showed the occurrence of thinning and perforations of the sinus mucosa in contact with the particles of the biomaterial. Despite the thinner width of the sinus mucosa of rabbits compared to that of humans, it might be assumed that over time, a progressive thinning of the mucosa and perforations might be also expected in humans. It was shown that the sinus mucosa tends to drive the particles that have created perforations out of the elevated space, with the perforation being sealed behind the particles by new mucosa [12]. However, some granules might be already incorporated into new bone so that the elimination of them would also include the resorption of that bone. This might gain more importance in case of the involvement of multiple granules of large dimensions considering that the perforations are most often associated with inflammatory infiltrates [12]. The outcomes from the present study suggest that it may be worthwhile for the use of biomaterials that cause limited injuries to the sinus mucosa over time to be taken into consideration.

## 5. Conclusions

In conclusion, the contact with bovine xenografts yielded thinning and perforations of the sinus mucosa. Despite the differences in characteristics and dimensions, no differences were found between the two xenografts in terms of the number of thinning mucosa sites and perforations. However, a trend of more events was found in the low-T compared to the high-T group.

## Figures and Tables

**Table 1:** Histometric data.

	2 Weeks			10 Weeks		
	low-T	high-T	*p* value	low-T	high-T	*p* value
Pristine mucosa in µm	61 ± 21	63 ± 14	0.617	62 ± 12	54 ± 8	0.107
Thinned mucosa in µm	26 ± 3.2	26 ± 5.3	0.789	19 ± 3.0	21 ± 4.5	0.065
No. sinus with thinned mucosa	8	9	>0.9999	10	10	NA
No. thinned mucosa zones	118	149	0.191	237	195	0.090
No. of sinuses with perforations	6	3	0.375	9	7	0.625
No. of perforations	8	3	0.188	19	14	0.898

**Table 2:** Number of sites of thinned mucosa categorized according to the width.

	<40	<30	<20	<10
2 weeks low-T (*n* = 9)	118	73	38	11
2 weeks high-T (*n* = 9)	149	98	58	16
10 weeks low-T (*n* = 10)	237	198	137	46
10 weeks high-T (*n* = 10)	195	148	96	44

## Data Availability

The data are available on reasonable request.
